# Transcriptional and epigenetic basis of Treg cell development and function: its genetic anomalies or variations in autoimmune diseases

**DOI:** 10.1038/s41422-020-0324-7

**Published:** 2020-05-04

**Authors:** Naganari Ohkura, Shimon Sakaguchi

**Affiliations:** Experimental immunology, Immunology Frontier Research Center, Osaka University, 3-1 Yamadaoka, Suita, Osaka 565-0871 Japan

**Keywords:** Autoimmunity, DNA methylation

## Abstract

Naturally arising regulatory CD4^+^ T (Treg) cells, which specifically express the transcription factor FoxP3 in the nucleus and CD25 and CTLA-4 on the cell surface, are a T-cell subpopulation specialized for immune suppression, playing a key role in maintaining immunological self-tolerance and homeostasis. FoxP3 is required for Treg function, especially for its suppressive activity. However, FoxP3 expression per se is not necessary for Treg cell lineage commitment in the thymus and insufficient for full Treg-type gene expression in mature Treg cells. It is Treg-specific epigenetic changes such as CpG demethylation and histone modification that can confer a stable and heritable pattern of Treg type gene expression on developing Treg cells in a FoxP3-independent manner. Anomalies in the formation of Treg-specific epigenome, in particular, Treg-specific super-enhancers, which largely include Treg-specific DNA demethylated regions, are indeed able to cause autoimmune diseases in rodents. Furthermore, in humans, single nucleotide polymorphisms in Treg-specific DNA demethylated regions associated with Treg signature genes, such as *IL2RA (CD25)* and *CTLA4*, can affect the development and function of naïve Treg cells rather than effector T cells. Such genetic variations are therefore causative of polygenic common autoimmune diseases including type 1 diabetes and rheumatoid arthritis via affecting endogenous natural Treg cells. These findings on the transcription factor network with FoxP3 at a key position as well as Treg-specific epigenetic landscape facilitate our understanding of Treg cell development and function, and can be exploited to prepare functionally stable FoxP3-expressing Treg cells from antigen-specific conventional T cells to treat autoimmune diseases.

## Introduction

Naturally occurring Treg (nTreg) cells, which constitute ~10% of CD4^+^ T cells in healthy individuals, constitutively express the transcription factor FoxP3 (in this review, FoxP3 is used to designate the protein in rodents and humans collectively) in the nucleus, CD25 and CTLA-4 on the cell surface as Treg function-associated molecules. They are indispensable for the maintenance of immunological self-tolerance.^1–3^ For example, removal of CD25^+^CD4^+^ Treg cells from the immune system results in spontaneous development of various autoimmune diseases, such as autoimmune thyroiditis and type 1 diabetes, in otherwise normal rodents.^4^ Mutations of the *FOXP3* gene cause similar autoimmune diseases in humans^5^ and systemic autoimmunity in mice.^5–7^ Mutations of the *IL2RA* and *CTLA4* genes also produce autoimmune/inflammatory diseases in humans and mice, at least in part, by impairing nTreg cell function. These findings on the vital roles of nTreg cells in immunological self-tolerance and homeostasis have facilitated recent studies on the molecular basis of Treg cell development and function. They have also prompted us to ask how genetic anomalies or variations in Treg development and function render the host susceptible not only to monogenic autoimmune diseases due to mutations of *FOXP3, IL2RA, CTLA4* and other genes affecting Treg cell function, but also to various polygenic common autoimmune diseases (such as type 1 diabetes and rheumatoid arthritis), which afflict ~10% of the population worldwide.^8^

A majority of nTreg cells present in the immune system are thymus-derived (thymus-derived Treg or tTreg cells). They are produced as a functionally mature and distinct T-cell subpopulation specialized for immune suppression, forming a cell lineage from the thymus to the periphery.^2^ A proportion of FoxP3^+^ Treg cells differentiate in the periphery from conventional T (Tconv) cells under certain conditions (peripherally derived Treg or pTreg cells).^9^ In addition, FoxP3^+^ Treg cells phenotypically similar to tTreg or pTreg cells can be generated in vitro (induced Treg or iTreg cells) from Tconv cells by antigen stimulation in the presence of TGF-β and IL-2.^10^ These Treg populations (tTreg, pTreg, and iTreg cells) commonly express FoxP3, CD25, CTLA-4 and other Treg function-associated molecules. It has been shown that tTreg and pTreg cells appear to be highly stable in the expression of FoxP3 and other Treg signature genes, hence sustaining stable suppressive function, while iTreg cells are apparently unstable and can be driven, under certain in vivo conditions, to differentiate into effector T cells.^11^ It needs to be elucidated then how nTreg cells acquire the Treg-specific gene expression pattern and its stability in the course of their physiological development in the thymus and the periphery and how the nTreg-like gene expression pattern and its stability can be conferred on iTreg cells for their clinical use.

In this review, we shall first review our current understanding of transcriptional and epigenetic basis of Treg cell development and function in the thymus, in particular, how FoxP3 expression and Treg-specific epigenetic changes (such as Treg-specific DNA hypomethylation, histone modifications and super-enhancer formation) distinctly or coordinately control Treg cell development and function. We shall then discuss the role of Treg cells in autoimmune diseases, especially how genetic anomalies such as *FOXP3* gene mutations or genetic variations such as autoimmune disease-associated single nucleotide polymorphisms (SNPs) revealed by genome-wide association study (GWAS) affect Treg cell development and function, being causative of autoimmune diseases. In addition, based on the transcriptional and epigenetic basis of nTreg cell development, we shall extend our discussion to how functionally potent and stable Treg cells can be generated from Tconv cells, especially from antigen-specific disease-mediating Tconv cells, for therapeutic purposes.

## FoxP3 and Treg-specific epigenome

### Is *FOXP3* the master control gene for Treg development and function?

The *FOXP3* gene mutations impair Treg function, especially suppressive activity; and ectopic expression of FoxP3 is able to confer suppressive function on Tconv cells.^12–14^ In addition, a majority of FoxP3^+^ nTreg cells appear to be highly stable in function for maintaining natural self-tolerance because inoculation of nTreg cells is able to effectively prevent autoimmune diseases in many animal models.^2^ Further, they are functionally stable despite their highly proliferative state under physiological conditions presumably due to their constant recognition of self-antigens or microbial antigens from commensal microbes.^15–17^ Because of the indispensable role of FoxP3 for Treg cell function, FoxP3 has been considered to be the master transcription factor for Treg cell function and its constitutive expression to be sufficient to sustain the function.

There are, however, many reports showing that FoxP3 expression alone is insufficient in Treg-type gene expression.^18–20^ For example, ~70% of the total gene expression differs between FoxP3-overexpressed Tconv cells and FoxP3^+^ nTreg cells. Some Treg signature genes such as *Ikzf2* (helios) and *Ikzf4* (Eos) are not expressed by the former.^20^ Conversely, FoxP3-deficient Treg cells isolated from *Foxp3* gene knockout mice show a gene expression pattern similar to that of FoxP3-intact normal nTreg cells.^18,21^ In humans, upon in vitro T-cell receptor (TCR) stimulation, Tconv cells can express FoxP3 transiently and at a low level, without exhibiting suppressive activity.^22,23^ Further, some FoxP3^+^ T cells naturally present in healthy humans and mice do not possess Treg suppressive activity but secrete inflammatory cytokines.^24,25^

**Fig. 1:**
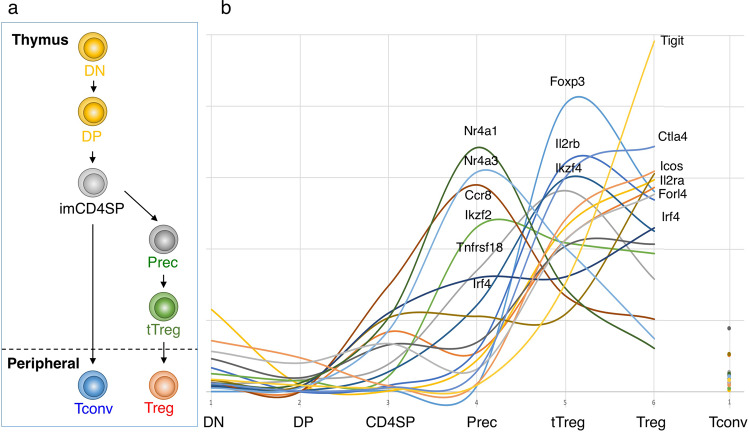
Expression of Treg-associated genes along Treg differentiation in the thymus. **a** Schematic representation of the developmental paths of Treg cells. DN: double negative T cells, DP: double positive T cells, imCD4SP: immature CD4 single positive T cells, Prec: Treg precursor cells, tTreg: thymic Treg cells, Tconv: conventional T cells. **b** The gene expression profile of the Treg-associated genes based on the deposited RNA-seq data.^26^ Relative expression level of each gene at each developmental stage is shown.

There is also accumulating evidence that FoxP3 expression per se is not required for Treg cell lineage commitment and their early differentiation in the thymus. For example, the expression of Treg-related genes has already begun prior to FoxP3 expression in the course of Treg cell development in the thymus. As revealed by time course profiling of Treg-related gene expression along tTreg cell differentiation (Fig. 1),^26^ the expression of Treg marker genes such as *Foxp3* and *Il2ra* is detected at a late tTreg cell differentiation stage. In contrast, *Nr4a* family genes play an important role in FoxP3 induction;^27,28^ Treg signature genes *Ikzf2*,^29,30^ *Ikzf4*,^31^ and *Tnfrsf18*,^32^ exhibit their expression peaks at the Treg precursor stage prior to FoxP3 induction; and Treg-specific histone modifications also occur before FoxP3 expression.^26^ In addition, FoxP3^−^CD25^+^CD4^+^ thymocytes readily give rise to FoxP3^+^ cells upon in vitro culture with IL-2 and TCR stimulation.^26^ These findings taken together indicate that cell fate decision of tTreg cells and their differentiation has already begun at a stage before the expression of the FoxP3 protein.

Thus, insufficiency of FoxP3 expression alone to confer full Treg function on mature Treg cells and FoxP3-independent cell commitment and differentiation of immature tTreg cells suggest the presence of a FoxP3-independent genetic mechanism controlling early tTreg cell differentiation as well as their function in the periphery.

### The role of Treg-specific epigenome for Treg cell development and function

Recent genome-wide studies have indeed revealed that FoxP3^+^ nTreg cells possess a number of unique transcriptional and epigenetic features, which appear to be acquired during Treg cell development and maintained throughout their lifespan. The comparison of the DNA methylation patterns of whole genomes between mouse Treg and Tconv cells has revealed that the genomic regions with different methylation status are present but very limited in number to ~300 regions (out of ~16,000 DNA methylated/hypomethylated regions assessed by MeDIP sequencing), constituting ~0.2% of methylated regions in the whole genome, and also limited to 0.5–2 kb long in the length of each region.^11^ The differences are mostly (>95%) Treg-specific CpG hypomethylation and not hypermethylation. They are preferentially located on gene bodies, particularly in intron 1 of Treg-upregulated genes, such as *Foxp3*, *Il2ra, Ctla4, Tnfrsf18*, and *Ikzf2*, but not in either the promoter regions or CpG islands, both of which are hardly different in methylation status between Treg and Tconv cells. In addition, such Treg-specific DNA hypomethylation correlates well with gene activation rather than repression.^11,33,34^ These observations collectively indicate that Treg-specific DNA demethylation at specific loci functions as an active enhancer for the transcription of the genes encoding Treg-specific functional molecules including FoxP3.

This formation of Treg-specific DNA hypomethylation is FoxP3 independent, as suggested by inability of FoxP3 overexpression to generate the hypomethylation in Tconv cells.^11^ Moreover, in FoxP3 mutant or gene-knockout mice, Treg-type gene expression, DNA hypomethylation, and histone modifications are specifically present in the majority of the Treg signature genes that are up-regulated in FoxP3-deficient tTreg cells as well as FoxP3-intact Treg cells.^11,18,26^ In mixed bone marrow chimeric mice generating both FoxP3-deficient and FoxP3-intact Treg cells without developing systemic inflammation, the former possess already in the thymus the Treg-type DNA hypomethylation not only at the *Foxp3* gene locus but also at other gene loci such as *Il2ra*, *Ctla4*, and *Tnfrsf18*.^11^ Notably, *Ikzf2* and *Ikzf4*, which cannot be expressed in Tconv cells by FoxP3 overexpression, are expressed in FoxP3-deficient Treg cells. It is also of note that the expression levels of these Treg function-associated molecules are slightly lower in FoxP3-deficient Treg cells compared with FoxP3-intact ones, suggesting a certain contribution of FoxP3 to up-regulation of the transcription of these genes, as further discussed below.

These results collectively suggest that Treg-specific DNA hypomethylation is developmentally generated in the genome of nTreg cells and heritable along their proliferation in the periphery, and that Treg-specific DNA demethylated regions (Treg-DRs) act as specific enhancers for up-regulation of Treg signature gene expression largely in a FoxP3-independent manner.

### Division of labor between FoxP3 and Treg epigenome

With FoxP3-independency of Treg-specific epigenetic changes, how do FoxP3 and the Treg-type epigenome, separately or coordinately, contribute to Treg-type gene expression? Comparison of the FoxP3-binding sites (~2900 detected by ChIP sequencing) with the Treg-DRs (~300 detected by MeDIP sequencing) has revealed that the two are not overlapping in the genome, except *Foxp3* conserved non-coding sequence 2 (CNS2).^11,34,35^ FoxP3-binding regions have no significant correlation with either up- or down-regulated genes in non-activated Treg cells.^34^ They are concentrated around the regions where chromatin state is commonly open in both Treg and Tconv cells,^36^ allowing FoxP3 to bind to the regions in FoxP3-overexpressed Tconv cells to exert its effects. Importantly, the genes possessing FoxP3-binding sites at promoter regions are strongly correlated with those genes (e.g., *Il2*, *Ifng*, and *Zap70*) which are transcriptionally repressed under TCR stimulated conditions, marked by H3K27me3, a repressive histone modification, and do not possess Treg-DRs.^11,34,37^ For example, the *Il2* and *Ifn*γ genes, which are down-regulated in nTreg cells, can be repressed in Tconv cells by FoxP3 overexpression alone without detectable Treg-specific epigenetic changes at these gene loci.^11^ FoxP3 is indeed able to actively repress gene transcription of key modulators of T cell activation and function by recruiting the HAT/HDAC complexes.^37,38^ In addition, upon stimulation by TCR or cytokines, FoxP3 is subjected to posttranslational modifications including acetylation, ubiquitination, and phosphorylation, and these FoxP3 modifications enhance the DNA binding ability of FoxP3 and thereby its transcriptional ability.^39–42^ These results collectively indicate that FoxP3 functions mainly as a transcriptional repressor under TCR stimulated conditions.

In contrast to the FoxP3-controlled genes described above, the genes associated with Treg-specific DNA hypomethylation are highly correlated with the genes (*e.g., Ikzf2*, *Ikzf4*, *Il2ra* and *Ctla4*), including *Foxp3*, that are up-regulated in unstimulated steady-state nTreg cells. Furthermore, Treg-DRs are found in the regions exhibiting open chromatin and H3K27ac modification in naïve Treg cells. These observations indicate that Treg-specific DNA hypomethylation, which allows binding of various transcription factors and chromatin conformation changes, contributes to maintaining constitutive gene expression of Treg signature genes in steady-state Treg cells including naïve Treg cells.

Thus, FoxP3 and Treg-DRs regulate Treg-specific gene expression in a complementary manner in steady and activated states, thereby achieving Treg-type gene expression and maintaining Treg functional activity and stability. It is also of note that, despite the expression of common proteins such as CD25 and CTLA-4 by Treg and activated Tconv cells, the epigenomic changes, in particular the Treg-specific DNA hypomethylation pattern, enable clear differentiation between Treg and activated Tconv cells at the genomic level, for example, at the *Il2ra* or *Ctla4* locus.^11^ The Treg-type DNA hypomethylation pattern is therefore a more specific marker than mere FoxP3 expression in defining functionally stable Treg cells especially when FoxP3 expression is induced or lost transiently.^11,43^

### Treg-specific super-enhancers and the genome organizer Satb1 for Treg cell differentiation

**Fig. 2:**
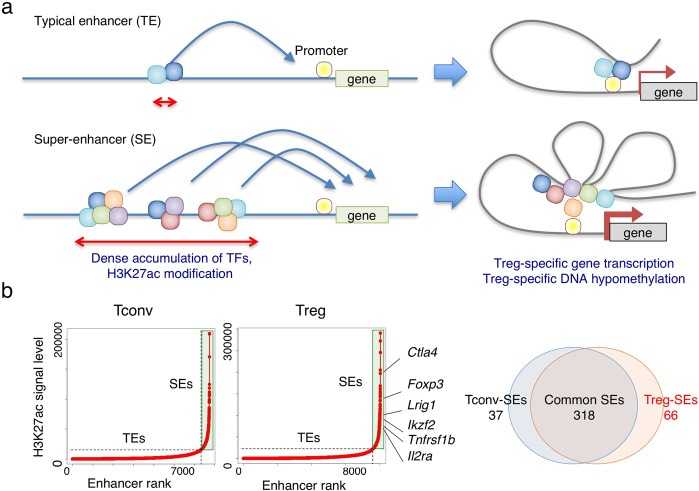
Treg-specific super-enhancers controlling Treg-specific transcription and epigenetic changes. **a** Super enhancers (SEs) defined as genomic regions with dense clustering of highly active enhancers accompanying strong activation-linked histone modification (such H3K27ac), promoted open chromatin states, strong binding of multiple transcription factors, and increased DNA demethylation. They contribute to the cell-type specific gene expression and cell lineage determination. **b** Treg-specific SEs are associated with Treg signature genes.

**Fig. 3:**
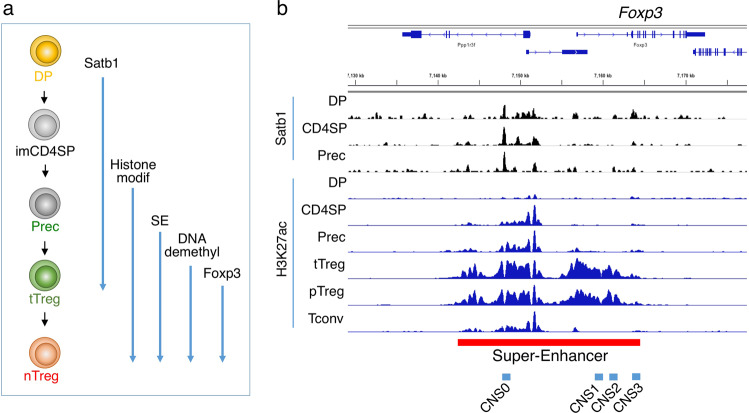
Establishment of Treg-specific super-enhancers along Treg differentiation in the thymus. **a** Developmental events in the genome along tTreg differentiation. Satb1: Satb1 expression, Histone modif: Treg-type histone modifications, SE: Treg-specific super-enhancers, DNAdemethyl: Treg-specific DNA demethylation, FoxP3 exp: FoxP3 expression. **b** Developmental changes in Satb1-binding and H3K27ac modification. The super-enhancer region at the *Foxp3* gene locus is indicated by bar.

Based on the critical roles of transcription networks with FoxP3 at a key position and Treg-specific epigenomic changes, as discussed above, it can be asked how each role can be triggered and integrated to drive Treg cell development and to control their function. A number of recent studies have identified cell type-specific super-enhancers (SEs), which are defined as genomic regions with dense clustering of highly active enhancers accompanying strong activation-linked histone modification, open chromatin states, strong binding of transcription factors, and increased DNA demethylation.^44^ Compared with typical enhancers, SEs have been shown to strongly activate the genes that define cell identity and determine cell lineage specificity.^45^ In fully differentiated nTreg cells, 66 Treg-specific SEs have been identified out of 384 SEs defined in the whole genome as the regions exhibiting strong H3K27ac signal assessed by H3K27ac ChIP sequencing (Fig. 2).^26^ Treg-specific SEs are indeed associated with Treg signature genes such as *Foxp3*, *Il2ra*, *Ctla4*, *Gitr*, and *Ikzf2*. They largely contain Treg-DRs.^46^ In the course of tTreg differentiation, activation of SEs occurs early at the CD4^+^CD8^-^ stage and becomes augmented through the Treg precursor to tTreg stage, accompanying increase in activation-related histone modifications such as H3K27ac, H3K4me3, and H3K4me1 as well as decrease in H3K27me3, a repression-related histone modification, at the SE regions (Fig. 3). The regions also show the binding of cohesin complexes such as CTCF and MED1, indicating that chromatin looping occurs between SEs and nearby gene promoters, leading to the gene activation. These changes are induced prior to the expression of associated Treg signature genes and in parallel in these gene loci,^26^ indicating that they are not passive changes following gene expression, but active changes necessary for the expression of the genes.

The genome organizer Satb1, which is specifically expressed in thymocytes,^47^ plays an essential role for establishing Treg-specific SEs. The Satb1 expression level is the highest in CD4^+^CD8^+^ double-positive (DP) and immature CD4 single-positive (imCD4SP) thymocytes and subsequently reduced. Binding of Satb1 is frequently observed, with slight accumulation of H3K27ac, in DP thymocytes in the genomic regions where SEs are established later in Treg differentiation.^26,47,48^ Notably, unlike typical transcription factors, Satb1-binding occurs in closed chromatin regions at the DP stage (Fig. 3). Furthermore, Satb1 deficiency from the DP stage on abrogates Treg-specific SE activation and Treg cell development and, as a consequence, induces severe autoimmune diseases.^26^ Satb1 and MLL4, an enzyme involved in enhancer priming, commonly occupy the newly identified conserved enhancer region, designated CNS0 at the *Foxp3* locus, with subsequent activation of the enhancers at CNS3 and CNS2, and then the promoter.^26,49^

**Fig. 4:**
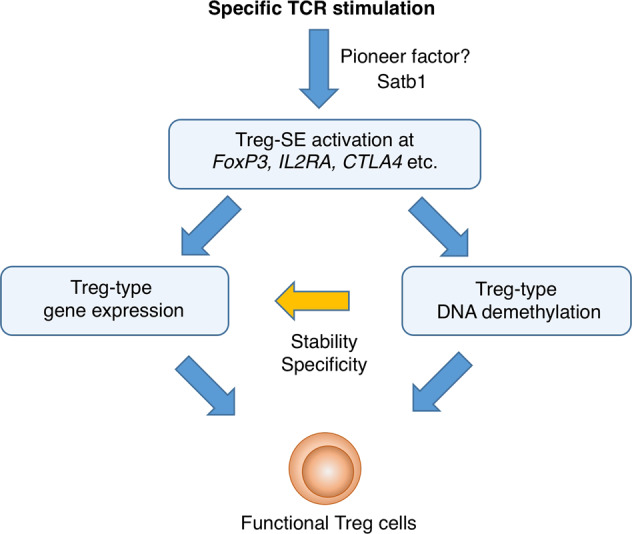
Treg-SEs control Treg-specific gene transcription and epigenetic changes in developing Treg cells. Treg cell development requires both the induction of Treg-specific epigenome and the establishment of Treg-type transcription networks including FoxP3 at a key position.

Thus, the establishment of Treg-specific SEs and its activation at Treg signature genes such as *Foxp3*, *Il2ra*, and *Ctla4* in parallel accompanies gradual opening of chromatin, an increase in permissive histone modifications, increased binding of critical transcription factors, and the generation of specific DNA hypomethylation at the SE regions, triggering and promoting Treg cell differentiation. The transcription complexes containing Satb1 may thus act as a “pioneer factor” for Treg-specific SE establishment and activation by first binding to inactive regions, changing the chromatin status from closed to open, and inducing a permissive state for gene transcription (Fig. 4).^26,35,50,51^

## Treg cell lineage determination and differentiation

With key contributions of FoxP3, Treg-specific epigeneome, and Treg-SEs to Treg cell development and function, it can be asked how these molecular events control Treg cell development in the thymus and the periphery as well as in vitro Treg generation from Tconv cells.

### Triggers of Treg cell development

At the stage of thymic Treg precursors as FoxP3^−^CD25^+^CD4^+^ thymocytes, both FoxP3 expression and Treg-specific DNA hypomethylation patterns have not been established. However, when these cells are cultured in the presence of IL-2 and TCR stimulation in vitro, they easily acquire these traits,^26^ indicating that Treg cell fate decision has already been made at or before the precursor stage. The initial event in the precursors can therefore be a clue for understanding a trigger for Treg cell development. The changes observed in the precursor stage are only low level H3K4me1 and H3K27ac histone modifications and Satb1-binding at the limited loci.^26^ The chromatin in these Satb1-bound regions gradually becomes open as the developmental stage proceeds; and the transcription factors important for FoxP3 induction, such as Runx1, Cbfb, Bcl11b, and Ets1,^52–57^ become binding to the regions. Foxo1 and Foxo3 transcription factors also contribute to the Foxp3 induction via their binding to the *Foxp3* locus.^58^ These changes are particularly prominent in Treg-specific SEs, with little changes in conventional or typical enhancers or promoters. The initial event for Treg-specific SE activation thus appears to involve TCR stimulation, Satb1-binding, IL-2 signaling, and binding of a set of transcription factors. In addition, specific transcription factors or chromatin remodeling factors that drive tTreg differentiation by binding to the SE regions remain to be characterized.

In contrast with tTreg cells, pTreg cells assume a different developmental process and require a different molecular trigger(s) for their differentiation.^59^ Tconv cells in the peripheral sites such as the intestinal mucosa can express FoxP3 by the exposure to cytokines, microbial or metabolic products, or TCR stimulation, or a combination of them.^60^ Many of these cells end up with transient FoxP3 expression, but some of them seem to acquire tTreg cell-like stable immunological characteristics including stable FoxP3 expression and Treg-type epigenome.^11,23,61^ For example, when Tconv cells are transferred to T-cell deficient mice, they give rise to some FoxP3^+^ T cells, which gradually acquire the Treg-type DNA hypomethylation pattern similar to the one seen in tTreg cells.^11^ The generation of pTreg cells does not require Satb1, a factor essential for tTreg generation and SE formation; Satb1 deficiency even facilitates pTreg cell generation.^26^ The result suggests that, in contrast with tTreg cells, FoxP3 induction can occur prior to the induction of Treg-specific DNA demethylation in pTreg cells, and might somehow contribute to their tTreg cell-like epigenome formation. It needs to be explored whether a common factor(s) or a signal(s) can trigger the formation of a Treg-specific epigenome in both tTreg and pTreg cells.

### Treg functional stability versus plasticity

Despite the requirement of functional stability of nTreg cells for their maintenance of self-tolerance, it is controversial whether they are functionally stable even at an inflammation site where inflammatory cytokines are abundant or, alternatively, they are functionally plastic and capable of differentiating into effector Tconv cells under certain circumstances.^62–64^ Since Treg cells appear to possess a TCR repertoire more skewed than Tconv cells to recognizing self-antigens, the plasticity of Treg cells to become converted to effector T cells may facilitate autoimmunity, as shown in some animal models.^63,65,66^ In addition, some reports have shown that prolonged exposure of Treg cells to Th1, Th2, or Th17 cytokines somehow results in the loss of immunosuppressive activity and the production of inflammatory cytokines.^67–69^ Cellular metabolites and metabolic signaling also affect the stability of FoxP3 in Treg cells, which predominantly depend on fatty-acid oxidation for their proliferation, differentiation and survival.^70,71^ In contrast, cell fate tracing by the use of FoxP3 fate reporter mice has demonstrated that nTreg cells are highly stable in function and phenotype under various circumstances.^64^ In addition, transcription of a reporter gene (*e.g*., eGFP) in Treg cells may affect endogenous FoxP3 expression and thereby impairs their function, rendering the host more susceptible to autoimmunity.^72^ Furthermore, several studies have shown that the apparent plasticity of Treg cells could be attributed to a subpopulation of FoxP3^+^ cells possessing incomplete Treg-type epigenome.^11,24^

Thus, taking into account the independency between FoxP3 expression and Treg-specific epigenome formation, it is most likely that FoxP3^+^ cells are heterogeneous in Treg-specific epigenome, including those with full Treg-type DNA hypomethylation and those without. The former are stably suppression-competent, constituting a majority of nTreg cells, and engaged in the maintenance of dominant self-tolerance, while the latter, which are present even in healthy individuals as a small fraction of FoxP3^+^ cells,^25,46^ may lose FoxP3 expression and convert to FoxP3^−^ Tconv cells under certain circumstances.^73^

### Transient FoxP3 expression by activated Tconv cells

Another confounding issue, in addition to the heterogeneity of FoxP3^+^ cells, is transient FoxP3 expression by activated Tconv cells especially in humans. Human naïve Tconv cells readily express FoxP3 at a low level upon stimulation with anti-CD3/CD28 antibodies in vitro and lose the expression after proliferation. Stimulation of mouse naïve T cells with TGF-β can also induce FoxP3 expression and such iTreg cells show a similar phenotype, such as CD25 and CTLA-4 expression, as nTreg cells.^16,74^ It has been shown, however, that nTreg cells and iTreg cells are not the same in functional stability, gene expression, and DNA methylation status of Treg signature genes.^11,75,76^ For example, nTreg cells are functionally more stable than TGF-β-induced iTreg cells, which can be mainly attributed to the differences in the epigenome, i.e., incomplete acquisition of Treg-type DNA hypomethylation by iTreg cells.^15^ Thus, since iTreg-inducing stimuli cannot confer on iTreg cells the stable and heritable epigenetic traits specific in nTreg cells, FoxP3 expression in iTreg cells is a “cell response” rather than “cell differentiation” of Tconv cells.

### Phenotypic and functional adaptability of Treg cells

As an additional confounding issue, Treg cells may adapt to their environment and substantially change their phenotype while maintaining their core suppressive function. For example, they are able to gain the expression of transcription factors and chemokine receptors normally associated with Th1,^77,78^ Th2,^79–81^ Th17,^82,83^ and Tfh cells,^84,85^ while they do not produce inflammatory cytokines because of FoxP3-dependent repression of the corresponding cytokine genes. These events enable Treg cells to express a particular Th-determining transcription factor and acquire the ability to migrate to the site of inflammation mediated by the corresponding Th cells.

In addition, Treg cells are resident in healthy tissues and engaged in not only immune suppression but also tissue repair and other non-immune functions, with gene expression profiles different from Treg cells in lymphoid organs. For example, Treg cells present in skeletal muscle produce the growth factor amphiregulin that acts to enhance the regeneration of muscle satellite cells and potentiate muscle repair.^86^ Those in visceral adipose tissue express the transcription factor PPARγ, and act to control obesity-associated inflammaition in the adipose tissue.^87,88^ Treg cells also massively accumulate in the mouse brain after ischaemic stroke, potentiating neurological recovery from ischaemic brain injury.^89^ It remains to be determined how genetically induced Treg core program composed of the transcription factor network (involving FoxP3 and other transcriptional regulators such as Bach2, IRF-4, Blimp1, and BATF, which control terminal differentiation of Treg cells)^90,91^ as well as Treg-specific epigenetic changes allow Treg cells to acquire these adaptive properties and functions beyond immune suppression.

## Autoimmune diseases and Treg cells

Genetic anomalies and environmental insults that affect Treg cells in number or function can be causative of autoimmune diseases.^2^ Similar to *FOXP3* mutations, mutations or anomalies of other genes expressed in immune cells may cause autoimmune diseases if they affect Treg cell development and function. These monogenic autoimmune/inflammatory diseases can be called “Tregopathies”.^92^ Furthermore, genetic variations such as SNPs found at gene loci of Treg function-associated genes can alter Treg cell function to various extents and thereby contribute to the development of polygenic common autoimmune diseases.

### Monogenic autoimmune diseases due to mutations of *FOXP3* and other genes

The IPEX (immune dysregulation, polyendocrinopathy, enteropathy, X-linked) syndrome due to *FOXP3* gene mutations is unequivocal evidence that deficiency or dysfunction of nTreg cells can be a primary cause of not only autoimmune diseases such as type 1 diabetes, but also inflammatory bowel disease and allergy in humans. Among seventy mutations of the *FOXP3* gene so far reported, the most frequent (~40%) mutations are present in the region encoding the C-terminal forkhead DNA-binding domain and abrogate the expression of functional FoxP3 protein, causing severe clinical manifestations.^93^ Other mutations also affect FoxP3 expression to various degrees with resulting variable phenotypes.

Mutations of Treg signature genes cause severe autoimmune diseases as seen in *FOXP3* mutations. For example, fatal autoimmune disease occurs in individuals with mutations of the *IL2RA*.^94^ Autoimmune/inflammatory disease also develops in family groups with heterologous mutations of CTLA-4, which hamper Treg expression of CTLA-4 in resting as well as activated states.^95,96^ The deficiency of LRBA (lipopolysaccharide-responsive and beige-like anchor protein), which is indispensable for CTLA-4 trafficking, causes severe autoimmunity due to loss of CTLA-4 expression by Treg cells.^97^ Other monogenic “Tregopathies” include inflammatory diseases due to *BACH2* and *STAT3* mutations, DiGeorge (22q11.2 deletion) syndrome, Omenn’s syndrome, and APECED (autoimmune polyendocrinopathy- candidiasis-ectodermal dystrophy) syndrome, which accompanies various organ-specific autoimmune diseases due to loss-of-function mutations of the *AIRE* gene and has decreased numbers of naïve Treg cells in the circulation.^98,99^

### Treg cells in multifactorial and polygenic autoimmune diseases

Since the discovery of an indispensable role of nTreg cells in immunological self-tolerance in humans as illustrated by IPEX syndrome, efforts have been made to search for functional or numerical anomalies, or variations, in nTreg cells in common autoimmune diseases.^100^ It is generally difficult, however, to determine whether an anomaly or variation found in Treg function or number is a cause or a consequence of an autoimmune response.

**Fig. 5:**
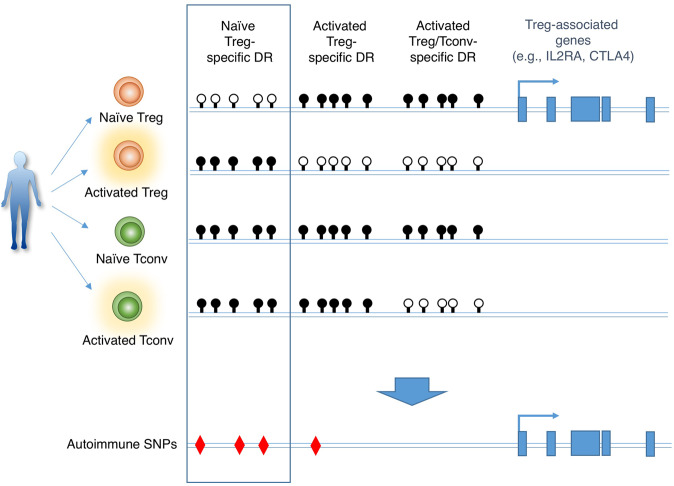
Location of autoimmune SNPs at Treg-DRs specific for naïve Treg cells. Characterization of specific demethylated regions in the genome of naïve or activated Treg or Tconv cells reveals that SNPs associated with autoimmune disease (e.g., SNPs found at *IL2RA* or *CTLA4* gene loci) are predominantly present at naïve Treg-specific DRs. Black and white circles represent methylated and demethylated CpGs, respectively.

Recent GWAS of common polygenic autoimmune diseases have revealed that ~60% of autoimmune-causal SNPs are mapped to non-coding enhancer regions of immune cells.^44,101,102^ For example, autoimmune SNPs are present in enhancer regions of T cell activation-associated genes such as *IL2RA* and *CTLA4*, which are also Treg function-associated.^103–105^ Yet it remains unclear whether such T cell-related autoimmune SNPs should affect the development and function of either autoimmune-causing Tconv cells or autoimmune-suppressing Treg cells. By profiling the epigenetic status of separate populations of Treg and Tconv cells in naïve and activated states, we have recently shown that autoimmune-associated SNPs are highly enriched in naïve Treg-specific DRs, next in activated Treg-specific DRs, but to a much lesser extent in activation-induced DRs common in Tconv and Treg cells (Fig. 5).^46^ Autoimmune SNPs are also enriched in Treg-specific SEs, which largely include Treg-DRs, but not in activation-specific SEs. The results indicate that autoimmune SNPs mapped to the genes associated with T-cell functions are predominantly associated with loss-of-function in Treg cells, rather than gain-of-function in effector Tconv cells, thereby rendering the host susceptible to common autoimmune diseases.

## Therapeutic application of Treg cells

### In vivo expansion of nTreg cells and Treg adoptive cell therapy

Given the capacity of Treg cells to induce stable immune tolerance, efforts have been made to expand or generate them or enhance their suppressive function to treat autoimmune and other immunological diseases. Due to their expression of the high affinity IL-2 receptor, Treg cells are highly sensitive to changes in IL-2 availability.^106,107^ Low doses of IL-2 are therefore under clinical trial to selectively expand Treg cells while avoiding complications from IL-2 signaling on NK cells or effector T cells. This approach has been demonstrated to successfully expand nTreg cells in patients with graft-versus-host disease (GvHD), type 1 diabetes, alopecia areata and HCV-induced vasculitis.^108^ In addition, since activated Treg cells express TNFR2 at much higher levels than activated Tconv cells, stimulation of TNFR2 by specific antibodies or agonists can preferentially expand nTreg cells in vivo and in vitro, and has been shown to be effective in treating GvHD.^109,110^

Treg-based adoptive cell therapy is another therapeutic approach to autoimmune diseases by purifying circulating Treg cells from a patient, expanding them in vitro by TCR stimulation in the presence of IL-2, and transferring them back to the patient. Naïve nTreg cells can be expanded 500–2000 fold, making it feasible to generate clinically usable numbers of Treg cells from limited quantities of blood.^111^ Transferred Treg cells reportedly persist in the recipients for at least one year, and phase 1 trials of autologous nTreg cell transfer have already demonstrated that this approach is feasible and safe in patients with type 1 diabetes.^112^ To make Treg adoptive cell therapy more efficacious, it needs to be determined which ways, antigen-specific Treg cells prepared by in vitro antigen stimulation or polyclonally expanded Treg cells with expectation of antigen-specific expansion after in vivo transfer, are therapeutically more effective. In addition, given their relative scarcity in human blood and their slow rate of in vitro expansion, better ways of purification and expansion need to be devised. A strategy to bypass these problems is to generate CAR-Treg cells which express antibody Fab region specific for a particular self-antigen and strongly suppress a particular autoimmune responses.^113^

### Conversion of Tconv cells into Treg cells

Besides therapeutic use of nTreg cells, iTreg cells generated by antigen stimulation of naïve Tconv cells in the presence of TGF-β and IL-2 has been extensively studied in experimental settings.^114^ In addition, activation of the AKT signaling pathway impairs the development of nTreg cells; and inhibitors of the pathway, for example, rapamycin can induce FoxP3 when combined with premature termination of TCR signaling.^115,116^ These iTreg cells in humans and mice, however, lack Treg-type epigenomic changes, especially Treg-specific DNA demethylation, hence functionally unstable, and are difficult to be generated from activated or effector T cells, or in the presence of inflammatory cytokines.

An ideal strategy for antigen-specific immune suppression is to convert not only naïve but also effector/memory Tconv cells mediating autoimmune disease into functionally stable FoxP3^+^ Treg cells in vivo and in vitro. Chemical inhibition of cyclin-dependent kinases (CDK) 8/19, which reversibly associate with the Mediator complex and control transcription positively and negatively, is able to induce FoxP3 in antigen-stimulated effector/memory as well as naïve CD4^+^ and CD8^+^ T cells in vitro.^117^ Furthermore, in vivo administration of a CDK8/19 inhibitor along with antigen immunization is able to generate functionally stable (i.e., epigenetically in Treg-type) antigen-specific FoxP3^+^ Treg cells, which effectively suppress autoimmune disease in animal models. The in vitro induction is dependent on STAT5 activation, independent of TGF-β, and not affected by inflammatory cytokines. The results indicate that a TCR-emanated signal through CDK8/19 is physiologically repressing FoxP3 expression in activated Tconv cells and that release from the repression suffices to induce FoxP3 in activated Tconv cells.

Thus, FoxP3 can be induced in Tconv cells by targeting distinct signaling pathways (e.g., TGF-β-SMADs, AKT-mTOR, and TCR-CDK8/19 pathways). A combination of targeting these pathways, together with the installation of Treg-type epigenome (see below), is envisioned to generate better iTreg cell preparations for adoptive Treg cell therapy.

### How can Treg-type epigenome be installed in iTreg cells?

As discussed above, mere expression of FoxP3 is not sufficient for iTreg cell production. Treg-specific epigenome, especially Treg-specific DNA hypomethylation, needs to be induced in iTreg cells.

TCR stimulation plays an important role in both FoxP3 induction and Treg-type epigenome formation; however, the quantity and quality required for the two events are likely to be different.^11,118,119^ For FoxP3 induction, the strength of TCR stimulation is important; and too strong or weak TCR stimulation fails to induce FoxP3.^120,121^ A moderate-intensity TCR stimulation by self-peptide/MHC rapidly induces FoxP3 in developing Treg cells in the thymus.^118^ In contrast, induction of Treg-specific DNA demethylation appears to require continuous TCR stimulation. In the thymus, FoxP3 expressing T cells and T cells that have acquired Treg-specific DNA demethylation are reportedly detected in different cell fractions.^122^ Continuous in vitro TCR stimulation of Tconv cells can partially induce Treg-type DNA demethylation.^11^ It is thus likely in the thymus that developing T cells receiving TCR stimulation at appropriate intensity and duration may differentiate into functionally stable tTreg cells with Treg-type epigenome. How co-stimulatory signals and cytokines contribute to this process remains to be determined for physiological tTreg cell development and for functionally stable iTreg cell induction in vitro.

## Conclusions and perspective

We have reviewed here recent understanding of how the networks of transcription factors including FoxP3 and Treg-specific epigenetic changes, such as DNA hypomethylation and histone modifications, contribute to the establishment and maintenance of Treg-specific gene expression in order to ensure Treg cells to be a functionally and phenotypically distinct T-cell subpopulation persisting in the periphery. Specific co-ordination between transcription factors and epigenetic alterations may activate specific SEs to initiate Treg cell differentiation at least in tTreg development. Furthermore, there is accumulating evidence that anomalies or alterations of Treg cell function and development significantly contribute to the development of various immunological and inflammatory diseases. In addition to monogenic autoimmune diseases such as IPEX syndrome due to *FOXP3* gene mutations, subtle genetic variations, such as SNPs, at enhancer regions of the genes encoding Treg function-associated molecules may affect Treg-specific gene expression to various extents, rendering the host susceptible to autoimmune diseases.

Based on these recent progresses in Treg cell research, it is hoped that future investigations will elucidate the following issues. First, how the Treg cell lineage is determined at an early stage, before FoxP3 expression, of tTreg differentiation via possible binding of a particular transcription factor(s) or epigenetic changes permissive to its binding, or a combination of these events; similarly, how the transcription factor network including FoxP3 and the epigenetic landscape together control pTreg differentiation, in particular what is common in these molecular events between tTreg and pTreg development; and how the Treg-specific transcription factor network and the Treg-specific epigenetic changes can be installed in iTreg cells for preparing functionally stable Treg cells.^123^ These studies will make Treg-based or -targeting immunotherapy to be a real therapeutic modality for autoimmune and other immunological diseases.
